# InAs/InAsSb Type-II Strained-Layer Superlattice Infrared Photodetectors

**DOI:** 10.3390/mi11110958

**Published:** 2020-10-26

**Authors:** David Z. Ting, Sir B. Rafol, Arezou Khoshakhlagh, Alexander Soibel, Sam A. Keo, Anita M. Fisher, Brian J. Pepper, Cory J. Hill, Sarath D. Gunapala

**Affiliations:** NASA Jet Propulsion Laboratory, California Institute of Technology, Pasadena, CA 91109, USA; Sir.B.Rafol@jpl.nasa.gov (S.B.R.); Arezou.Khoshakhlagh@jpl.nasa.gov (A.K.); Alexander.Soibel@jpl.nasa.gov (A.S.); Sam.A.Keo@jpl.nasa.gov (S.A.K.); Anita.M.Fisher@jpl.nasa.gov (A.M.F.); Brian.J.Pepper@jpl.nasa.gov (B.J.P.); Cory.J.Hill@jpl.nasa.gov (C.J.H.); Sarath.D.Gunapala@jpl.nasa.gov (S.D.G.)

**Keywords:** InAs/InAsSb, type-II superlattice, infrared detector, mid-wavelength infrared (MWIR), unipolar barrier

## Abstract

The InAs/InAsSb (Gallium-free) type-II strained-layer superlattice (T2SLS) has emerged in the last decade as a viable infrared detector material with a continuously adjustable band gap capable of accommodating detector cutoff wavelengths ranging from 4 to 15 µm and beyond. When coupled with the unipolar barrier infrared detector architecture, the InAs/InAsSb T2SLS mid-wavelength infrared (MWIR) focal plane array (FPA) has demonstrated a significantly higher operating temperature than InSb FPA, a major incumbent technology. In this brief review paper, we describe the emergence of the InAs/InAsSb T2SLS infrared photodetector technology, point out its advantages and disadvantages, and survey its recent development.

## 1. Introduction

The II-VI semiconductor HgCdTe (MCT) is the most successful infrared photodetector material to date. MCT grown on nearly lattice-matched CdZnTe (CZT) substrate offers continuous cutoff wavelength (λ_cutoff_) coverage from the short-wave infrared (SWIR) to the very long wavelength infrared (VLWIR), while providing a high quantum efficiency (QE) and low dark current for high-performance applications. In general, III-V semiconductors are more robust than their II-VI counterparts due to their stronger, less ionic chemical bonding. III-V semiconductor-based infrared focal plane arrays (FPAs) excel in operability, spatial uniformity, temporal stability, scalability, producibility, and affordability. InGaAs FPAs with λ_cutoff_ ~ 1.7 μm perform at near theoretical limit and dominate the SWIR FPA market. InSb FPAs (λ_cutoff_ ~ 5.3 μm), despite a significantly lower operating temperature than MCT, lead the mid-wavelength infrared (MWIR) market in volume due to their superior manufacturability and lower cost. The major limitation for traditional bulk III-V semiconductor detectors grown on (nearly) lattice-matched substrates is the lack of pervasive cutoff wavelength adjustability.

### 1.1. Advances in III-V Infrared Material

One method to achieve a wide-range cutoff wavelength adjustability in III-V semiconductors is to use bulk InAsSb alloy grown on metamorphic buffers. Recent results show that the InAsSb band gap bowing is significantly larger than previously believed, and up to λ_cutoff_ ~ 12.4 μm could be achieved [1]. In addition, nearly lattice-matched or pseudomorphic III-V semiconductor type-II superlattices (T2SLs) can provide a high degree of flexibility in cutoff wavelength. They can have a sufficient absorption strength to attain ample quantum efficiency, are less susceptible to band-to-band tunneling than bulk semiconductors [2], and are capable of achieving reduced Auger generation-recombination in properly designed structures [3].

III-V semiconductor extended SWIR (eSWIR) detectors are commonly grown on either InP or GaSb substrates. The first group includes the well-known extended InGaAs [4] and lattice-matched [5] and strain-compensated [6] InGaAs/GaAsSb type-II quantum wells. The second includes bulk GaInAsSb [7,8] and InPSb [9], as well as InAs/GaSb [10], InAs/GaSb/AlSb/GaSb [11], InAs/AlSb [12], InAs/InSb/AlSb/InSb [13], and InAs/InAsSb/AlAsSb [14] superlattices.

For MWIR and long wavelength infrared (LWIR) detectors, InAs/GaSb and InAs/InAsSb are the two most common T2SL absorbers used. The former is well-established, and has been described in detail in various review articles [15,16,17]. The latter [18,19,20] emerged more recently as an alternative with simpler growth [18], better defect tolerance, and longer minority lifetime [21], but smaller cutoff wavelength range, weaker optical absorption [22,23], and more challenging growth-direction hole transport [24,25]. In Section 3, we will discuss some basic properties of the InAs/InAsSb T2SLS in more detail.

### 1.2. Unipolar Infrared Detector Architecture

The unipolar barrier infrared photodetector architecture is now widely recognized as a highly effective platform for developing high-performance infrared photodetectors, as exemplified by the nBn [26], the XBn [27,28], the complementary barrier infrared detector (CBIRD) [29,30], the double heterostructure (DH) [31,32], and the pMp [33]. A unipolar barrier blocks one carrier type (electron or hole) but allows the unimpeded flow of the other. The unipolar barrier photodetector architecture can be used to lower generation-recombination (G-R) dark current by suppressing Shockley–Read–Hall (SRH) processes [26], and has also been used to reduce surface-leakage dark current [26,34,35] in devices with n-type absorbers. This has been especially beneficial for III-V semiconductor-based infrared photodiodes, many of which traditionally tend to suffer from excess depletion dark current and a lack of good surface passivation. Unipolar barrier infrared photodetectors have been successfully implemented for a variety of bulk and superlattice absorbers.

### 1.3. Antimonide Barrier Infrared Detectors

Taking advantage of the recent developments in the antimonide bulk and type-II superlattice infrared absorber material and the advances in the unipolar barrier infrared detector architecture, a new generation of infrared detectors has been successfully implemented in a variety of cutoff wavelengths ranging from SWIR to LWIR. In this section, we briefly mention a few examples to illustrate the effectiveness and versatility of this approach.

The 2006 paper by Maimon and Wicks on the nBn infrared detector [26] has been one of the most influential works in the field of infrared photodetectors in recent years. The performance of the nBn is enhanced by a specially constructed barrier that blocks the majority but not the minority carriers. These unipolar barriers, which are heterostructure barriers that can block one carrier type (electron or hole) but allow the un-impeded flow of the other, can be used to suppress the G-R dark current and surface leakage current [26,34,35], which are the two main dark current mechanisms that have plagued III-V semiconductor infrared detectors. The initial nBn devices used either InAs absorber grown on InAs substrate, or lattice-matched InAs_0.91_Sb_0.09_ alloy grown on GaSb substrate, with cutoff wavelengths of ~3.2 and ~4 µm, respectively. These nBn detectors could operate at much higher temperatures than InSb-based MWIR detectors, although their spectral responses do not cover the full 3 to 5 μm MWIR atmospheric transmission window like InSb detectors.

**Figure 1:**
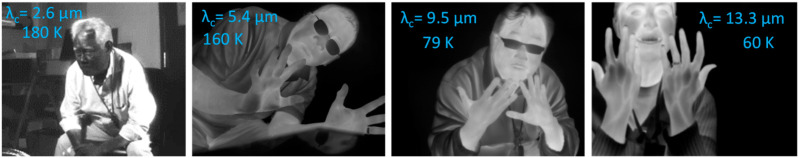
Images from extended-SWIR (λ_c_ = 2.6 μm), MWIR (λ_c_ = 5.4 μm), LWIR (λ_c_ = 9.5 μm), and VLWIR (λ_c_ = 13.3 μm) FPAs made from antimonide bulk and T2SL unipolar barrier infrared detectors.

The antimonide barrier infrared detector concept has also been successfully implemented in the eSWIR using a GaInAsSb quaternary absorber and an AlGaSb or AlGaAsSb unipolar electron barrier [7,8]. By adjusting the alloy composition of an GaInAsSb infrared absorber, its cutoff wavelength can vary from ~1.8 to ~4 μm while remaining lattice-matched to the GaSb substrate. In addition, for a lattice-matched GaInAsSb absorber of a given composition, a matching AlGaAsSb electron unipolar barrier can be found for building an nBn (or XBn) detector. The left panel of Figure 1 shows an image taken with an eSWIR FPA made from such an nBn detector at the NASA Jet Propulsion Laboratory (JPL), with a cutoff wavelength of ~2.6 μm at 180 K.

The desire to develop high-performance nBn detectors with cutoff wavelength capable of covering the full 3 to 5 μm MWIR atmospheric transmission window (like InSb) motivated the exploration of T2SL nBn detectors. MWIR InAs/GaSb T2SL nBn detectors were demonstrated by Rodriguez et al. in 2007 [36], soon after the original nBn work by Maimon and Wick [26]. More recently, the InAs/InAsSb type-II strained-layer superlattice (T2SLS) has emerged as a highly versatile absorber material. Along with matching AlAsSb or AlGaAsSb electron barriers (typically lightly p-doped), the InAs/InAsSb type-II T2SLS has been used very effectively in implementing MWIR and LWIR unipolar barrier infrared detectors. Figure 1 shows images of FPAs fabricated at JPL from InAs/InAsSb T2SLS unipolar barrier infrared detectors using cutoff lengths of 5.4, 9.5, and 13.3 μm. In the next section, we will describe the InAs/InAsSb T2SLS infrared detectors in more detail.

## 2. The Emergence of InAs/InAsSb T2SLS Infrared Photodetectors

### 2.1. Historical Background

Though much less explored than the InAs/Ga(In)Sb T2SLS, the development of InAs/InAsSb/InSb (Gallium-free, or Ga-free) T2SLS for infrared emitter and detector applications has a long and interesting history that predates the InAs/Ga(In)Sb T2SLS detector [37]. The InAsSb/InAsSb T2SLS was originally proposed by Osbourn as a means of achieving in III-V semiconductors a smaller bandgap than bulk InAsSb in order to reach longer wavelengths [38,39]. This is made possible because of the type-II band alignment (which can lead to a superlattice band gap that is smaller than its constituent bulk materials) and strain-induced band-gap reduction. Researchers at Sandia set out to implement this idea using the InAsSb/InSb SLS grown on InSb substrate with an InSb-rich composition-graded InAsSb strain-relief buffer [40]. By 1990, S. R. Kurtz and co-workers reported a number of photodiodes and photoconductors with cutoff wavelengths ranging from 8.7 to beyond 10 µm [41,42,43], thus validating the concept of the LWIR InAsSb T2SLS detector. In 1995, Zhang reported continuous-wave operation 3.3–3.4 µm midinfrared lasers based on InAs/InAsSb T2SLS emitter grown on InAs substrate [44]. Meanwhile, researchers in the UK also explored the “As-rich” InAs/InAsSb T2SLS grown metamorphically on GaAs substrates. In 1995, Tang and colleagues from the Imperial College reported InAs/InAsSb T2SLSs with a strong photoluminescence (PL) intensity and infrared emission ranging from 4 to 11 µm [45]. Working with other UK research groups, it was found that As-rich InAs/InAsSb T2SLS with band gaps comparable to InSb have substantially suppressed Auger processes when compared to InSb [46]. Because of the room-temperature Auger suppression, it was suggested that this material may be attractive for mid-IR diode laser applications. In 1999, Pullin and co-workers from the Imperial College demonstrated the room-temperature operation of mid-IR light-emitting diodes based on the InAsSb T2SLS [47].

In the decade since 2000, developments in InAs/Ga(In)Sb T2SLS [2,48] flourished while research activities in the Ga-free InAsSb T2SLS were relatively dormant. Signs of renewed interest in InAsSb T2SLS appeared in 2009, when researchers from Simon-Fraser published a paper on the growth and optical characterization of InAs/InAsSb T2SLS structures strain-balanced relative to the GaSb substrate [49]. The interest in this material as an infrared detector absorber grew stronger as the Zhang Group and collaborators reported significantly longer LWIR minority carrier lifetimes in InAs/InAsSb T2SLS than in the InAs/GaSb superlattice [21]. In 2012, the Zhang Group demonstrated an InAs/InAsSb T2SLS LWIR photodetector [19] based on the nBn device design. The Razeghi Group provided further impetus by showing the versatility of the InAs/InAsSb T2SLS, having reported LWIR [20,50,51], very long wavelength infrared (VLWIR) [52], and bias-selectable dual-band mid-wavelength infrared (MWIR)/LWIR dual-band infrared photodetectors [53]. While the Zhang Group focused on LWIR Ga-free T2SLS, the NASA Jet Propulsion Laboratory started working on Ga-free T2SLS in 2008 in an effort to develop mid-wavelength barrier infrared detectors capable of covering the full 3 to 5 µm MWIR atmospheric transmission window [54]. This effort culminated in the demonstration of InAs/InAsSb T2SLS base mid-wavelength high operating temperature barrier infrared detectors in 2010 and FPAs in 2011 [18,54], and with the subsequent development of detectors and FPAs with longer cutoff wavelengths (see Figure 1) [55].

### 2.2. Mid-Wavelength InAs/InAsSb T2SLS Barrier Infrared Detectors

Perhaps the most significant impact that the InAs/InAsSb T2SLS has had thus far is in the demonstration of MWIR detectors and FPAs. The MWIR FPA market is traditionally dominated in volume by InSb, with a smaller number of MCT FPAs (with higher operating temperature) filling the needs for high-performance applications. According to G. Fulop of Maxtech International, in 2018 InSb accounted for over 50% of the photodetector-based infrared FPA market (in number of units, all cutoff wavelengths). InAs/InAsSb T2SLS unipolar barrier infrared detector-based FPAs have demonstrated that, like MCT, they can operate at a much higher temperature than their InSb counterparts, all while retaining the same III-V semiconductor manufacturability advantages. The concept and initial detector and FPA results of the InAs/InAsSb T2SLS high operating temperature (HOT) barrier infrared detector (BIRD) were first described in patent documents [18]. The details were reported subsequently in the literature [56,57]; here, we briefly mention some key results.

**Figure 2:**
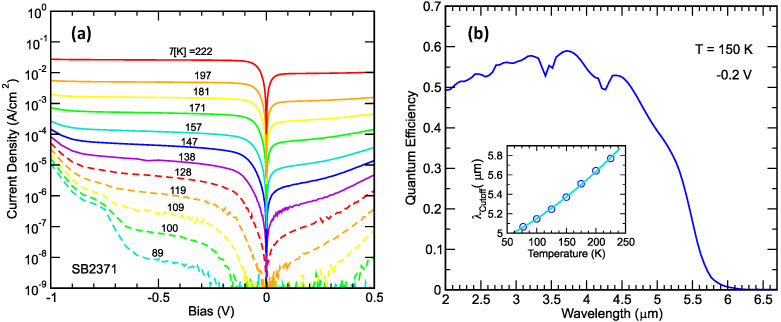
(**a**) Dark current density–voltage characteristics for an InAs/InAsSb type-II strained-layer superlattice nBn detector taken at temperatures ranging from 89 to 222 K. (**b**) Back-side illuminated spectral quantum efficiency (QE) for the same detector without anti-reflection coating, taken under a −0.2 V bias at 150 K. The inset shows the 50% peak QE cutoff wavelength as a function of temperature.

Figure 2a shows the detector dark current density as a function of applied bias for detector temperatures ranging from 89 to 222 K for an nBn device with an InAs/InAsSb T2SL absorber and an AlAsSb electron unipolar barrier grown on GaSb substrate. Figure 2b shows the spectral quantum efficiency of the detector, demonstrating the full coverage of the MWIR transmission window. The inset shows the dependence of the detector 50%-peak-QE cutoff wavelength as a function of temperature. At 150 K, the 50% cutoff wavelength is 5.37 µm, and the quantum efficiency at 4.5 µm is ~52% without anti-reflection coating. In general, the dark current performance is quite good. Under a −0.2 V bias, the dark current density at 157K is 9.6 × 10^−5^ A/cm^2^, which a factor ~4.5 higher than that predicted by the MCT Rule 07 [58] for a cutoff wavelength of 5.4 µm. At 150K, the dark-current-limited and the f/2 black-body (300 K background in 3–5 µm band)-specific detectivities are, respectively, 4.6 × 10^11^ and 3.0 × 10^11^ cm-Hz^1/2^/W.

The detector material was used to fabricate 24-µm pitch, 640 × 512 format arrays hybridized to the SBF-193 readout integrated circuit (ROIC). The cutoff wavelength is ~5.4 μm, closely matching that of the InSb FPA. A 160 K two-point corrected image taken with a resulting FPA is shown in Figure 1. At 160 K, the 300 K background, f/2 aperture mean noise equivalent differential temperature (NEDT) is 18.7 mK, with a standard deviation of 9.2 mK and a NEDT operability of 99.7%. The ion-implanted planar InSb FPAs and molecular beam epitaxy (MBE)-grown epi-InSb FPAs typically operate at 80 K and 95–100 K [59], respectively. The InAs/InAsSb T2SLS-based mid-wavelength high operating temperature barrier infrared detector (HOT-BIRD) FPA has demonstrated significant operating temperature advantages over InSb. For 300 K background imaging applications, the mid-wavelength HOT-BIRD essentially combines the higher operating advantage of MCT with the III-V semiconductor material robustness advantages of InSb, thus firmly establishing the InAs/InAsSb T2SLS as a viable infrared FPA technology.

## 3. Basic Properties of the InAs/InAsSb T2SLS

In this section, we describe some basic properties of the InAs/InAsSb T2SLS and discuss its advantages and disadvantages as an infrared detector absorber material.

### 3.1. InAs/InAsSb T2SLS Electronic Properties

**Figure 3:**
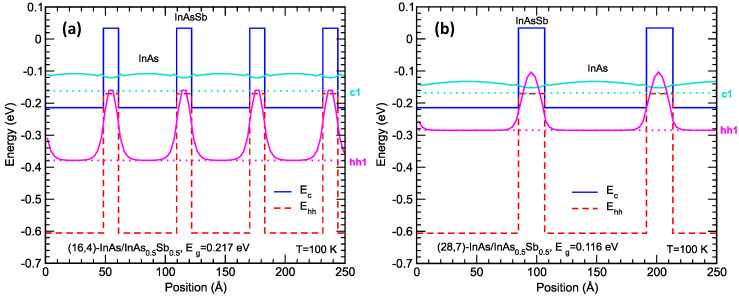
Energy band diagrams showing the bulk InAs and InAsSb conduction band edge (*E*c) and valence band edge (*E*_hh_), the superlattice zone-center c1 and hh1 energy levels (dotted lines), and the corresponding c1 and hh1 state probability densities (solid lines) for (**a**) the (16,4)-InAs/InAs_0.5_Sb_0.5_ strained-layer superlattice (SLS), and (**b**) the (28,7)-InAs/InAs_0.5_Sb_0.5_ SLS at 100 K. The InAs and InAsSb layers are, respectively, under tensile and compressive strain on the GaSb substrate.

Figure 3 shows the energy band diagrams for two (*m*,*n*)-InAs/InAs_0.5_Sb_0.5_ superlattices (*m* and *n*, respectively, being the number of monolayers of InAs and InAsSb in each superlattice period) that are strain-balanced with respect to the GaSb substrate. For a given layer thickness ratio (*m*/*n*), the InAsSb alloy composition is selected to achieve strain balancing. Varying the superlattice period (*m* + *n*) changes the band gap and the corresponding cutoff wavelength (λ_cutoff_ = *hc*/*E*_g_). In such a strain-balanced superlattice, typically the InAs layer is under slight tensile strain while InAsSb is under a relatively high compressive strain. Therefore, a comparatively thick InAs layer is required for strain balance against the thinner InAsSb layer. The InAs and InAsSb layers are, respectively, electron and hole quantum wells. Because the InAsSb electron barriers separating the InAs electron quantum wells are relatively weak, the c1 (lowest superlattice conduction band state) wavefunction is only weakly confined, as can be seen in the c1 probability density plots in Figure 3. On the other hand, it can also be seen in Figure 3 that the hh1 (heavy-hole 1, the highest superlattice valence band state) wavefunction is substantially localized in the InAsSb valence band quantum wells, which are separated by relatively thick layers of InAs hole barriers. This is reflected in the corresponding band structure, which is discussed next.

**Figure 4:**
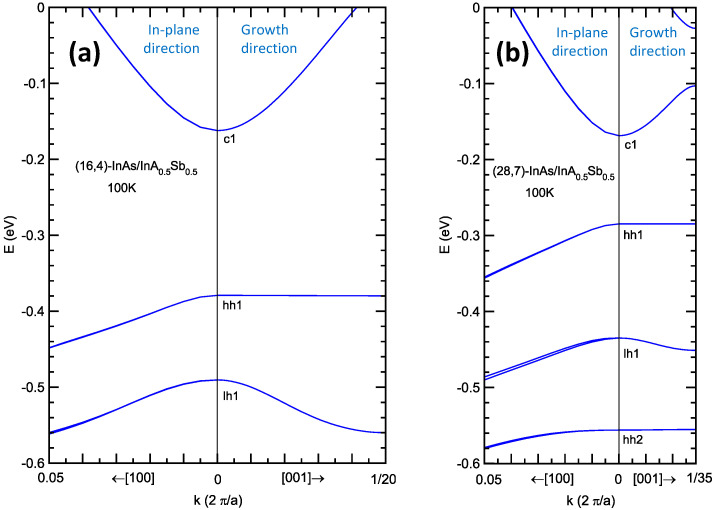
Energy band structure along an in-plane direction, and along the growth direction for (**a**) the (16,4)-InAs/InAs_0.5_Sb_0.5_ strained-layer superlattice (SLS) (*E*g = 0.217 eV; λ_cutoff_ = 5.7 µm), and (**b**) the (28,7)-InAs/InAs_0.5_Sb_0.5_ SLS (*E*g = 0.116 eV; λ_cutoff_ = 10.7 µm), on GaSb substrate at 100 K.

Figure 4 shows the energy band structures for the same two superlattices shown in Figure 3: (a) MWIR superlattice (16,4)-InAs/InAs_0.5_Sb_0.5_ with *E*_g_ = 0.217 eV and λ_cutoff_ = 5.7 µm, and (b) LWIR superlattice (28,7)-InAs/InAs_0.5_Sb_0.5_ with *E*_g_ = 0.116 eV and λ_cutoff_ = 10.7 µm. The relatively weak c1 state confinement and the stronger hh1 state confinement are reflected in the band structure, with the c1 subband having a strong dispersion along the growth direction, while the hh1 subband is nearly dispersionless along the growth direction. This indicates that, along the growth direction, electron transport is more favorable than hole transport (even more so than in the typical bulk semiconductors). The splitting between the hh1 and lh1 (light-hole 1) band is favorable for suppressing band-to-band tunneling, which depends on the c1-lh1 gap [2,37]. For the LWIR superlattice in Figure 4b, the hh1-lh1 splitting is actually larger than the c1-hh1 superlattice band gap. This is favorable for Auger-7 suppression [3]. For a more detailed discussion on the effect of superlattice band structure on infrared absorbers, see, for example, Reference [17].

**Figure 5:**
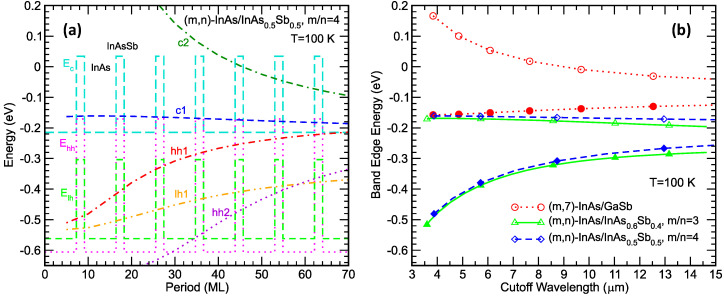
(**a**) The calculated c2, c1, hh1, lh1, and hh2 energy levels for a set of (4*n*,*n*)-InAs/InAs_0.5_Sb_0.5_ superlattices, plotted as functions of superlattice period in monolayers (MLs). The background shows a relevant superlattice energy band diagram, with an arbitrary horizontal length scale. (**b**) The calculated superlattice conduction band edge (*E*_c1_, open symbols) and valence band edge (*E*_hh1_, solid symbols) as functions of the cutoff wavelength for sets of (*m*,7)-InAs/GaSb, (3*n*,*n*)-InAs/InAs_0.6_Sb_0.4_ and (4*n*,*n*)-InAs/InAs_0.5_Sb_0.5_ superlattices.

Figure 5a shows how the calculated c2, c1, hh1, lh1, and hh2 energy levels vary in strain-balanced (*m*,*n*)-InAs/InAs_0.5_Sb_0.5_ superlattices, *m*/*n* = 4, as functions of *P*, where *P* = (*m* + *n*) is the superlattice period in units of monolayers. To help visualize the location of the superlattice energy levels, the background of Figure 5 shows a superlattice energy band diagram with the InAs and InAsSb strained conduction, heavy-hole, and light-hole band edges. Note that, since all the superlattices here have the same (*m*/*n*) ratio and therefore have the same energy band diagram except for a horizontal-axis scaling factor, the background band diagram in Figure 5 is shown with an arbitrary horizontal distance scale and can therefore be shared by all the calculated structures. As the superlattice period increases, the hh1 level rises while the c1 level stays relatively constant at just a few tens of meV above the InAs conduction band edge. The relatively constant c1 level can be exploited for constructing heterostructures with aligned conduction bands. For instance, an MWIR superlattice can be used as a unipolar hole barrier for an LWIR superlattice. As the superlattice period increases, the (c1-hh1) band gap decreases while the hh1-lh1 splitting increases. As mentioned earlier, a hh1-lh1 splitting larger than the c1-hh1 band gap is favorable for Auger-7 suppression. Figure 5b replots the c1 and hh1 levels, now as functions of the cutoff wavelength calculated from the superlattice band gap. To this, we add also the results for a set of strain-balanced (*m*,*n*)-InAs/InAs_0.6_Sb_0.4_ superlattices with (*m*/*n*) = 3, and a set of (*m*,7)-InAs/GaSb superlattices. The c1 levels for the two sets of InAs/InAsSb superlattices are both approximately independent of the cutoff wavelength and have approximately the same value. For the (*m*,7)-InAs/GaSb superlattices, the valence band edge remains approximately constant, since we fix the GaSb hole quantum well width at seven monolayers. In general, the conduction band edges of the InAs/InAsSb superlattices are low compared to those for the InAs/GaSb superlattices.

### 3.2. InAs/InAsSb T2SLS Advantages

**Figure 6:**
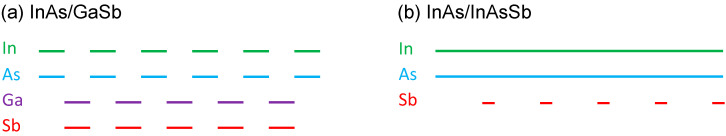
Schematic illustration of mechanical shutter sequences used in growing (**a**) InAs/GaSb and (**b**) InAs/InAsSb superlattices.

The InAs/InAsSb T2SLS has some advantages over the more established InAs/GaSb type-II superlattice (T2SL). The InAs/InAsSb T2SLS is easier to grow, has longer minority carrier lifetimes, and appears to have a better defect tolerance. Figure 6 illustrates the molecular beam epitaxy (MBE) shutter sequence used in the growths of InAs/GaSb T2SL and InAs/InAsSb T2SLS. In principle, the growth of the InAs/InAsSb T2SLS involves only opening and closing the Sb shutter, while the In and As shutters can stay open throughout [18]. This compares to the need to the use of four shutters in the InAs/GaSb T2SL. The growth of the InAs/GaSb T2SL is actually considerably more complicated than indicated in the simplified illustration of Figure 6, which does not include the strain-balancing interfaces required to achieve a high material quality. Thus, in general, the InAs/InAsSb T2SLS is simpler to grow.

InAs/InAsSb T2SLS has demonstrated longer minority carrier lifetimes than the InAs/GaSb T2SL [21,60,61]. For instance, the MW InAs/GaSb T2SL minority carrier lifetime has been reported at ~80 ns [62], while non-intentionally doped MW InAs/InAsSb T2SLS has exhibited minority carrier lifetime values ranging from 1.8 [61] to 9 µs [60], with a Shockley–Read–Hall (SRH) lifetime of ~10 μs [61].

There has also been evidence suggesting that the longer minority carrier lifetime of InAs/InAsSb T2SLS is related to its defect tolerant. Tang et al. pointed out in their 1995 work [45] that, despite the high threading dislocations expected in the InAs/InAsSb SLSs grown with metamorphic buffers on highly lattice-mismatched GaAs substrates, the Shockley–Read contributions to recombination rates were low, as indicated by the strong photoluminescence (PL) intensity observed. It was hypothesized that this is due to the fact that defect state energy levels in the InAs/InAsSb T2SLS are resonant with the conduction band, rather than in the band gap where they could contribute to carrier recombination. The idea that the defect energy levels are in the conduction band was confirmed in recent pressure-dependence PL experiments on an MWIR InAs/InAsSb T2SLS grown on GaSb [63]. The reason for this is that the InAs/InAsSb T2SLS conduction band edge, like that for bulk InAs, is low, as can be seen in the theoretical results in Figure 5b. At JPL, we have seen anecdotal evidence for defect tolerance. One of the earliest MWIR InAs/InAsSb T2SLS nBn detector wafers was grown on a vintage 2011 developmental 4-inch-diameter GaSb substrate. At that time, the 4-inch substrate surface polishing was not nearly as mature as it is today. The fact that we were nevertheless able to obtain reasonable FPA results can be attributed in part to the defect tolerance of the InAs/InAsSb superlattice (the nBn device architecture being another major contributing factor) [54].

### 3.3. InAs/InAsSb T2SLS Challenges

The disadvantages of the InAs/InAsSb T2SLS compared to the InAs/Ga(In)Sb T2SLS are (1) weaker LWIR absorption, and (2) more challenging LWIR hole transport. Both are the results of the fact that for the InAs/InAsSb T2SLS a longer superlattice period is required in order to reach the same LWIR band gap as the InAs/Ga(In)Sb T2SLS.

**Figure 7:**
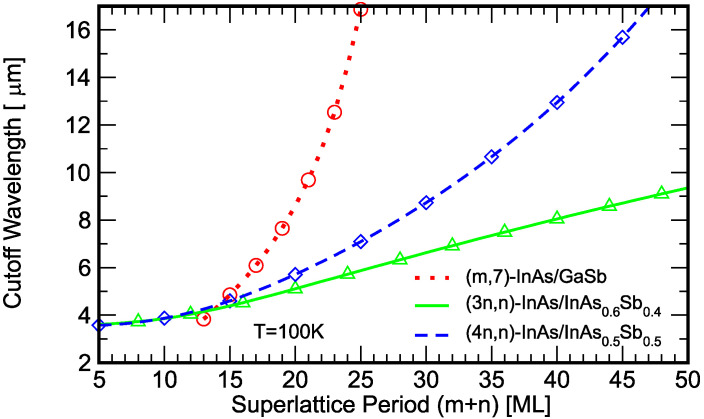
Calculated cutoff wavelength for the sets of (*m*,7)-InAs/GaSb, (3*n*,*n*)-InAs/InAs_0.6_Sb_0.4_, and (4*n*,*n*)-InAs/InAs_0.5_Sb_0.5_ superlattices, as functions of the superlattice period in monolayers (MLs).

Figure 7 shows the calculated cutoff wavelength as a function of superlattice period for the same three sets of superlattices discussed in Figure 5b. The cutoff wavelength is derived from the calculated superlattice band gap using the relationship λ_cutoff_ [µm] = 1.24/*E*_g_ [eV]. In the MWIR range, the three set of superlattices have comparable periods. As the cutoff wavelength increases, the periodicity advantage of the InAs/GaSb T2SL becomes more pronounced. Comparison between the two set of (*m*,*n*)-InAs/InAsSb superlattices show that the set with higher (*m*/*n*) ratio and higher Sb fraction InAsSb alloy is more favorable. In a type-II superlattice, the band-edge electron and hole wavefunctions are localized in different layers (see Figure 3). A longer superlattice period reduces the electron-hole wavefunction overlap, leading to weaker oscillator strength and smaller absorption coefficient. Early theoretical analysis by Grein et al. [64] showed that, compared to the InAs/GaInSb superlattice, the InAs/InAsSb superlattice requires wider InAs layers to achieve a comparable band gap and therefore produces smaller optical matrix elements; the calculated absorption coefficients for a 11 µm cutoff InAs/GaInSb T2SLS and a 10 µm cutoff InAs/InAsSb T2SLS are 2000 and 1500 cm^−1^, respectively. More recently, Vurgaftman et al. calculated the absorption coefficients for LWIR superlattices with band gaps of ~0.1 eV (corresponding to cutoff wavelengths of λ_cutoff_ = 10−12 µm), and showed that the InAs/InAsSb T2SLS absorption coefficient is approximately half as large as that for the InAs/GaSb T2SL [23]; at λ = 8 µm, the absorption coefficients are ~1250 and ~700 cm^−1^ for the InAs/GaSb (70 Å period) and the InAs/InAsSb (125 Å period) superlattices, respectively. Klipstein et al. modeled the dependence of the LWIR superlattice detector spectral quantum efficiency (QE) on the diffusion length, and concluded that, even for a very large hypothetical diffusion length, the InAs/InAsSb T2SLS has a significantly lower QE than the InAs/GaSb T2SL because of its weaker absorption coefficient [22].

The longer period of the LWIR InAs/InAsSb T2SLS also results in larger growth-direction hole conductivity effective masses, which in turn lead to lower vertical hole mobility and shorter diffusion length. From the textbook expressions for diffusion length ($L=\sqrt{D\tau_{r}}$), diffusivity ($D=\mu k_{B}T/e$), and mobility ($\mu =e\tau_{c}/m^{**}$), we see that the diffusion length depends explicitly on the conductivity effective mass through the expression $L_{i}={\left[\left(k_{B}T/m_{i}^{**}\right)\tau_{r}\tau_{c,i}\right]}^{1/2}$, where $m_{i}^{**}$ is the conductivity effective mass, $\tau_{r}$ is the minority carrier recombination lifetime, $\tau_{c,i}$ is the collision (momentum relaxation) time, and *i* is the direction index. It can be seen that a large growth-direction conductivity effective mass reduces diffusion length, which in turn limits the practical absorber thickness. In the case of LWIR InAs/InAsSb T2SLS, which has s weaker absorption coefficient than the corresponding InAs/GaSb T2SL and bulk absorbers, the inadequate absorber thickness limits the attainable quantum efficiency. Calculations by Klipstein et al. demonstrate from another perspective the strong QE dependence on the hole diffusion length in an LWIR InAs/InAsSb T2SLS XBn detector: for a 9.7 µm cutoff detector with a fixed 5 µm thick n-type absorber, the QE at 8.5 µm is 40% for a 5 µm hole diffusion length but drops to only 10% for a 1 µm hole diffusion length [22].

**Figure 8:**
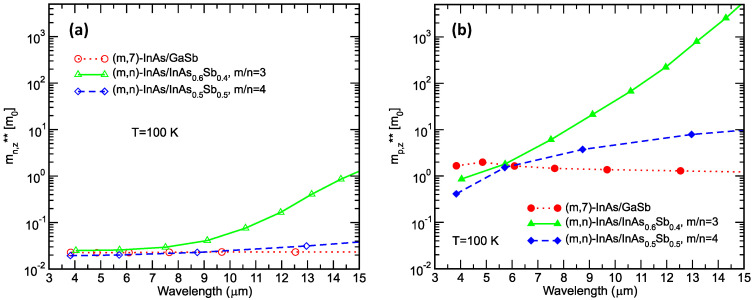
Growth-direction (**a**) electron and (**b**) hole conductivity effective masses for two families of InAs/InAsSb superlattices, and a set of InAs/GaSb superlattices.

Figure 8 shows the calculated electron and hole conductivity effective masses along the growth direction (*m*_n,z_** and *m*_p,z_**, respectively) as functions of the cutoff wavelengths for the same sets of superlattices discussed in Figure 7. The conductivity effective masses are thermally averaged quantities which take into consideration the anisotropy and non-parabolicity in the superlattice band structure; detail discussions can be found in References [24,25]. Figure 8a shows the calculated growth-direction electron conductivity effective mass *m*_n,z_**. For the InAs/GaSb and the (*m*/*n*) = 4 InAs/InAs_0.5_Sb_0.5_ superlattices, *m*_n,z_** values are quite small since the c1 wavefunctions are only weakly confined in the relative shallow conduction band quantum wells separated by thin InAsSb barriers (see Figure 3). For (*m*/*n*) = 3 InAs/InAs_0.6_Sb_0.4_ superlattices (with lower Sb fraction InAsSb), the electron effective mass *m*_n,z_** can increase with the cutoff wavelength to rather large values because of the much longer superlattice period; see Table 1 for a comparison of the superlattice period and *m*_n,z_** for three different superlattices, all with cutoff wavelengths in the 12–13 µm range. Figure 8b shows the calculated growth-direction hole conductivity effective mass *m*_p,z_** for the same three sets of superlattices. Again, the *m*_p,z_** values for the three sets of superlattices are very similar in the MWIR. However, as the cutoff wavelength increases, the superlattice periods required to reach the same cutoff wavelength diverge (see Figure 7), and the *m*_p,z_** for the three sets of superlattices also diverge significantly (see Table 1 for specific examples). For the InAs/InAsSb superlattices, *m*_p,z_** can be very large when the InAsSb hole quantum wells are separated by a wider InAs layer (see Figure 3). Here, the higher Sb fraction in the InAsSb alloy decreases the InAs/InAsSb T2SLS period, and is therefore especially helpful in reducing the growth-direction hole conductivity effective masses. Since the growth-direction electron conductivity effective masses *m*_n,z_** are much smaller than the hole effective masses *m*_p,z_**, the diffusion length can be much longer in the p-type InAs/InAsSb T2SLS than in the n-type, which would be more favorable for achieving a higher quantum efficiency. However, for reticulated detector structures with p-type InAs/InAsSb T2SLS absorbers, the exposed side-wall surfaces are inverted to degenerate the n-type (like in InAs) and passivation is often needed for reducing the surface related dark currents; for a discussion on surface leakage dark current mechanisms in unipolar barrier detectors, see Reference [34,65,66,67]. Despite the challenges, (V)LWIR n-type and p-type InAs/InAsSb T2SLS detectors with cutoff wavelengths ranging from 8 to 15 µm and front-side illuminated quantum efficiencies from 2.5% to 40% have been reported [19,20,50,51,52,68,69].

### 3.4. Concepts for Addressing LWIR InAs/InAsSb T2SLS Challenges

LWIR and VLWIR InAs/InAsSb T2SLS have relatively long superlattice periods and therefore have relatively weak optical absorptions and short hole diffusion lengths, which are challenging for achieving a high quantum efficiency. We recently explored theoretically some ideas for addressing the challenges for the LWIR InAs/InAsSb superlattices [70]; here, we briefly summarize the results. In comparing the (*m*/*n*) = 3 InAs/InAs_0.6_Sb_0.4_ and the (*m*/*n*) = 4 InAs/InAs_0.5_Sb_0.5_ superlattices in the discussions above, we found that increasing the Sb fraction in the InAsSb alloy can reduce the InAs/InAsSb superlattice period significantly. In fact, at sufficiently high Sb fraction (~75%), InAs/InAsSb can match InAs/GaSb in terms of the superlattice period required to reach a given cutoff wavelength [70]. However, high Sb fraction InAs/InAsSb superlattices are more prone to Sb segregation [71,72,73,74], which can negate the period-reduction benefit of high-fraction Sb. Polytype superlattices [75] such as the “W” [31], “M” [76], and “N” [77] structures have been used for improving the oscillator strength over the basic InAs/GaSb T2SL. The analogous polytype W, M, and N superlattices formed by inserting thin AlAsSb barrier layers in InAs/InAsSb T2SLS have been considered as a means for increasing electron-hole wavefunction overlap for stronger optical absorption. This strategy turns out to be unfavorable because the presence of the AlAsSb barriers leads to increased band gap, and therefore increases the superlattice period required to reach a given cutoff wavelength [70]. Metamorphic growth on virtual substrates with larger lattice constants than GaSb can decrease the superlattice period needed to reach a specified cutoff wavelength, but this benefit should be weighed against the need for metamorphic buffer growth and the resulting higher defect density [70]. Finally, as mentioned previously, p-type InAs/InAsSb T2SLS has a longer diffusion length than n-type, but reticulated detector pixels with exposed p-type absorber side-wall surface would require passivation to suppress the surface leakage dark current.

## 4. Recent Development and Outlook

InAs/InAsSb T2SLS infrared detectors have been under very active development in recent years, with reported results by research groups worldwide in MWIR [18,56,57,78,79,80,81,82], LWIR [19,20,50,51,83,84], and VLWIR [52,85] detectors, as well as in MWIR/LWIR [53] and LWIR/VLWIR [68] bias-switchable dual-band detectors. Microlens [85] and resonant cavity structures [86] have been used to enhance the photo-response of InAs/InAsSb T2SLS infrared detectors. Studies of transport properties [87,88,89,90] and defect levels [63] have led to an improved understanding of InAs/InAsSb superlattices. While most of the more recent InAs/InAsSb T2SLS structures have been grown on (100) GaSb substrates by MBE, growths by a variety of modes have also been demonstrated. MWIR and LWIR detectors have been grown by metal organic chemical vapor deposition (MOCVD) [91,92,93,94]. Growths on GaAs [84], Si [80,95], Ge-Si [96], and AlSb (via metamorphic buffer on GaSb) [97] substrates have been reported. The growth of MWIR and LWIR detectors on (211)A and B, and (311)A and B GaSb substrates have also been demonstrated [69,98].

The MWIR InAs/InAsSb T2SLS FPA rivals InSb in manufacturability and affordability, but offers a 40 to 50 K higher operating temperature advantage, which can lead to a lower cryocooler size, weight, and power (SWaP). As such, it is poised to replace the InSb FPA, a major incumbent technology, in many imaging applications. The MWIR InAs/InAsSb T2SLS detectors and FPAs have also demonstrated very low dark current densities at lower temperatures [99], and are therefore suitable for more demanding applications such as the CubeSat-based hyperspectral imaging of 300 K scenes while operating in an intermediate temperature range (100–120 K) [100]. Although LWIR InAs/InAsSb T2SLS FPAs have only achieved a moderate quantum efficiency, their demonstrated large-format capability and high uniformity and operability makes them already suitable for applications such as LWIR imaging for Earth remote sensing applications, where photon flux is abundant. Dual-band FPAs are also expected to find applications because of the manufacturability and cost effectiveness of InAs/InAsSb T2SLS FPAs. The further development of InAs/InAsSb T2SLS infrared detectors will continue to benefit from the infrastructure established largely during the the VISTA Program [101,102], including the availability of large-diameter format GaSb substrates [103,104,105,106] and multi-wafer growth capability at commercial foundries [107,108].

## Figures and Tables

**Table 1:** Growth direction electron and hole conductivity effective masses in units of bare electron mass for three superlattices with approximately the same cutoff wavelength.

Superlattice	Period (*m* + *n*) [monolayer]	λ_cutoff_ [µm]	*m*_n,z_** [*m*_0_]	*m*_p,z_** [*m*_0_]
(16,7)-InAs/GaSb	23	12.5	0.0233	1.29
(54,18)-InAs/InAs_0.6_Sb_0.4_	72	12.0	0.166	222
(32,8)-InAs/InAs_0.5_Sb_0.5_	40	13.0	0.0313	7.89
